# Posterior segment findings in a patient with immunotactoid glomerulonephritis

**DOI:** 10.3205/oc000023

**Published:** 2015-01-16

**Authors:** Aditi Gupta, Rangarajan Venugopal Prabhu, Amit K. Patel, Ramesh Sivaraj

**Affiliations:** 1 Department of Ophthalmology, Birmingham and Midland Eye Centre, Dudley Road, Birmingham, United Kingdom; 2 Heart of England NHS Foundation Trust, Sutton Coldfield, United Kingdom

**Keywords:** choroidal folds, cloudy cornea, immunotactoid glomerulonephritis

## Abstract

**Purpose:** To present a case with posterior segment findings in a patient with cloudy corneas secondary to immunotactoid glomerulonephritis (ITG).

**Methods:** A 57-year-old female was known to have bilateral cloudy corneas diagnosed 12 years ago secondary to immunotactoid glomerulonephritis. Clinically, fundus examination was difficult to visualise due to the density of her corneal opacities.

**Results:** B-scan ultrasound revealed significant retino-choroidal & non-inflammatory scleral thickening. The macula also showed signs of thickening in both eyes. Optical coherence tomography (OCT) showed thinning of the inner retinal layers and significant choroidal folds in both eyes. Electrodiagnostic tests (EDT) concluded loss of retinal ganglion cells with preservation of retinal function in both eyes.

**Conclusion:** This case widens the spectrum of findings seen in patients diagnosed with Immunotactoid Glomerulonephritis and alerts us to undertake detailed posterior segment examination where possible. Ocular coherence tomography (OCT) and B-scan ultrasonography are important adjuvants to help assess the posterior segment in patients with corneal opacities secondary to ITG.

## Introduction

Corneal involvement in patients diagnosed with immunotactoid glomerulonephritis (ITG) is well described in literature. ITG is characterised by deposition of abnormal monoclonal protein in patients with paraproteinemia and lymphoproliferative disorders. We present interesting posterior segment findings in a patient with cloudy corneas secondary to immunotactoid glomerulonephritis.

## Case description

A 57-year-old female was referred to the corneal clinic with a one-week history of seeing a ‘black spot’ in front of her right eye. Her past ocular history revealed bilateral cloudy corneas diagnosed 12 years ago secondary to ITG. She was reviewed on an annual basis but as she was asymptomatic with visual acuity 6/6 (LogMAR 0.0) both eyes and the appearance of her corneas had remained unchanged over seven years; she was discharged back to primary care. Her medical history revealed ITG following a renal biopsy carried out 20 years ago. 6 years ago, she was diagnosed with end stage renal failure and was on haemodialysis and continuous ambulatory peritoneal dialysis. She was also known to suffer with haemolytic anaemia and hypertension.

**Figure 1:**
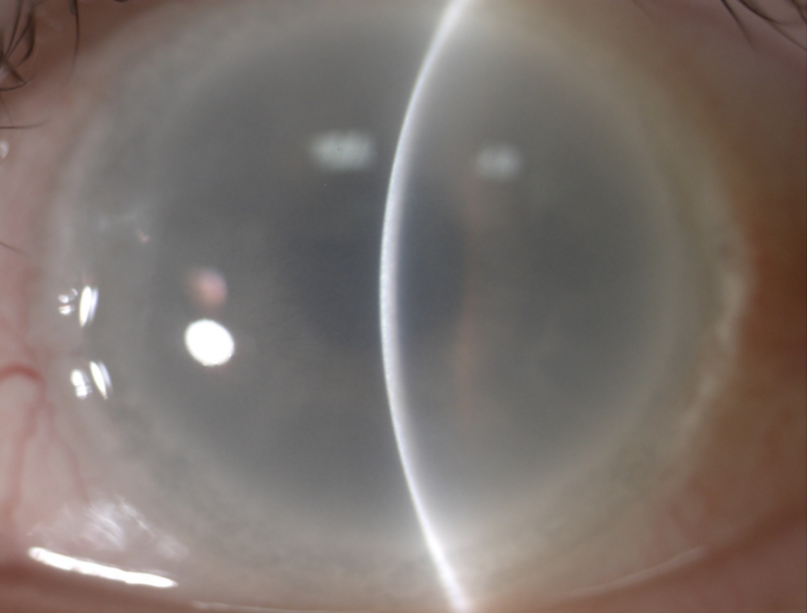
Slit-beam disclosing full thickness limbal to limbal opacification of the cornea

On examination, her best-corrected visual acuity was recorded as 6/7.5 (logMAR 0.2) and near vision N6 in both eyes. Corneal examination revealed full thickness limbal to limbal opacification in both eyes (Figure 1 (Fig. 1)). The central corneal thickness measured 611 microns and 608 microns respectively and intraocular pressures using Goldmann applanation tonometry were within normal range. There were no significant lens opacities seen although visualisation of the anterior and posterior segment was severely limited by the corneal changes. Perusing through her previous ophthalmic records, it was noted that fundus was difficult to visualise due to the density of her cloudy corneas and no particular findings were recorded. We decided to carry out optical coherence tomography (OCT) to assess her fundus and based on the OCT findings, B-scan ultrasound (USG) and electrodiagnostic tests (EDT) were performed.

**Figure 2:**
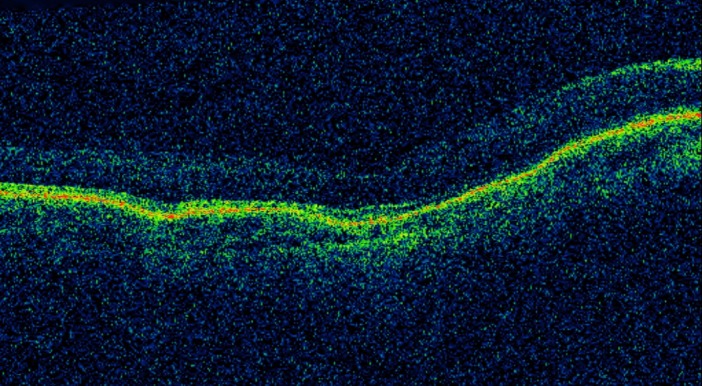
OCT scan is hazy due to media haze but both eyes showed thinning of the inner retinal layers and significant choroidal folds in both eyes

**Figure 3:**
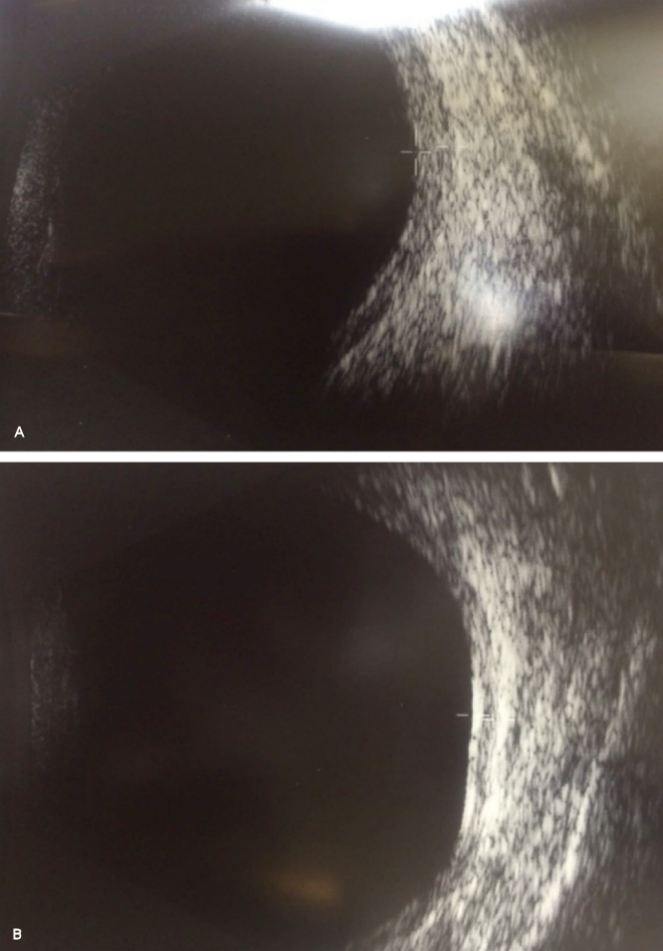
B-scan USG showing retino-choroidal thickening which is maximum temporally and scleral thickening with the absence of T-sign. In general, retinal contour was noted to be irregular.

OCT examination showed a hazy view but thinning of the inner retinal layers and choroidal folds was noted in both eyes. Photoreceptor layer appeared to be intact including the outer nuclear layer (Figure 2 (Fig. 2)). B-scan USG revealed significant overall scleral thickening and retino-choroidal thickening in both eyes, which was maximal temporally (Figure 3 (Fig. 3)). The maximum thickness of the macula was 2.1 mm in both eyes. There was no evidence of choroidal effusion or any sign of inflammation in the vitreous. Electroretinograms (ERGs) were well preserved to rod and cone stimulation with slight reduction of oscillatory potentials. There was a slight delay of the cone b-wave in the right eye. Pattern ERGs were reduced in both eyes. The test concluded loss of retinal ganglion cells with preservation of retinal function in both eyes.

In both eyes, visual evoked potential (VEP) to flash stimulus was essentially normal. Her visual field testing showed non-specific defects and Ishihara colour vision test was normal. 

The patient refused penetrating keratoplasty due to the risks attached but elected to consider this option at a later stage. We decided to observe this patient on a regular basis and carry out serial OCT scans. The patient was asked to report any changes to her vision. At 6-month follow-up, patient was asymptomatic and visual complaints of seeing a ‘black spot’ in her right eye resolved completely.

## Discussion

Immunotactoid glomerulonephritis (ITG) is a rare renal deposition disease and the diagnosis is based on analysis of renal biopsies. The primary differential diagnosis of this condition includes membranoproliferative or mesangiocapillary glomerulonephritis (MPGN). Both these glomerular diseases typically present with nephrotic syndrome. In ITG, the exact mechanism is unknown but deposition of immunoglobulin, particularly IgG κ and λ light chains and complement, suggests an immune system dysfunction. MPGN is a group of immune complex-mediated disorders characterized by subendothelial and mesangial deposition of immune complexes. Most of these patients progress to develop end stage renal failure. 

Corneal involvement in various systemic paraproteinemias or monoclonal gammopathies is well established and various morphological characteristics mimicking hereditary and degenerative corneal disorders have also been described [1], [2]. There is evidence that some of these patients with paraprotenemias have tubular glomerular deposits characteristic of ITG [3]. Immunohistological tests have also confirmed immunoglobulin deposition in various layers of the cornea. Garibaldi et al. [2] described corneal IgG kappa deposits similar to microtubular deposits seen in renal biopsy proven immunotactoid glomerulonephritis thereby proposing the term immunotactoid keratopathy. Most patients present with minimal visual symptoms and require no specific ocular therapy. In the very few cases of severe and lasting corneal involvement, penetrating keratoplasty seems to be the treatment of choice [3].

The choriocapillaris, Bruch’s membrane and RPE complex have been proven to have similar morphological characteristics as the glomerulus and there is histopathological evidence that immune complexes can get deposited at these sites [4], [5]. Most of this evidence has been described in Type II MPGN. Clinically, fundus findings simulate drusen-like deposits with mottled pigmentation [5]. Deb Coville et al. [6] described an interesting case where the patient developed widespread retinal atrophy in Type II MPGN and recommended a ten-year follow-up by ophthalmologists on these patients to prevent permanent visual impairment, as these patients are known to have a greater incidence of diffuse retinal pigment alterations, pigment epithelial detachment and choroidal neovascularization. In a study conducted by McAvoy et al. [7], the authors describe a patient who lost vision as a result of possible choroidal neovascular membrane (CNV) diagnosed with type II MPGN and also suggested a possible correlation between the development of retinal changes and the duration of renal disease.

In our patient, renal biopsy showed deposits of IgG kappa and C3 at the tips of some glomerular loops and based on electron microscopy, a diagnosis of ITG was made. Subsequently, our patient developed generalized full thickness cloudiness of both her corneas. As her visual acuity continued to remain stable and the patient was asymptomatic, the need for detailed fundus examination was overlooked and patient was discharged to primary care. Her recent presenting complaint of ‘black spot in vision’ did not correlate with her cloudy corneas and it was then that the patient underwent an OCT scan. Choroidal folds seen on OCT both eyes are presumably a result of retino-choroidal & scleral thickening as confirmed by B-scan. We feel that this is an immune related process related to ITG as there were no signs of intraocular inflammation & ‘T-sign’ was absent on B-scan. One could argue that choroidal thickness is possibly altered as a result of chronic renal failiure and following haemodialysis but evidence shows that the average choroidal thickness decreases significantly in such patients [8] whereas choroidal thickness is notably increased in our patient. Immunopathological evidence is undoubtedly required to study the pathogenesis of immune complex deposition in the posterior segment of these patients. Loss of retinal ganglion cells confirmed on EDT correlates with thinning of the inner retinal layers seen on OCT imaging in both eyes. These findings further speculate a possible neurodegenerative effect as a result of related duration of the disease and effect of immune complex deposition and antibody dependent cytotoxicity. Larger studies are needed to explain potential retinal neuropathy in ITG. Transient visual dysfunction experienced by the patient only in the right eye is difficult to explain but perhaps related to her posterior segment findings. This is only a hypothesis as she was asymptomatic in her left eye and posterior segment findings are analogous in both eyes. Due to significant media opacities, fundus fluorescein angiogram was not carried out. However, this patient continues to remain under regular monitoring for any further changes. 

## Conclusions

To our knowledge, this is the first case describing retino-choroidal & scleral thickening in a patient with ITG. This case widens the spectrum of findings seen in patients diagnosed with ITG and alerts us to undertake detailed posterior segment examination in patients with corneal opacities. Corneal changes might not be the only cause of visual symptoms in patients with ITG. OCT and B-scan USG are useful tools for posterior segment assessment in these patients. This case also highlights the need for further studies on a cohort of patients diagnosed with ITG to assess their posterior segment findings.

## Notes

### Competing interests

The authors declare that they have no competing interests.
